# Comparison of Body Mass Index (BMI), Body Adiposity Index (BAI), Waist Circumference (WC), Waist-To-Hip Ratio (WHR) and Waist-To-Height Ratio (WHtR) as Predictors of Cardiovascular Disease Risk Factors in an Adult Population in Singapore

**DOI:** 10.1371/journal.pone.0122985

**Published:** 2015-04-16

**Authors:** Benjamin Chih Chiang Lam, Gerald Choon Huat Koh, Cynthia Chen, Michael Tack Keong Wong, Stephen J. Fallows

**Affiliations:** 1 Department of Family and Community Medicine, Khoo Teck Puat Hospital, Singapore, Singapore; 2 Saw Swee Hock School of Public Health, National University of Singapore, Singapore, Singapore; 3 Department of Clinical Sciences & Nutrition, University of Chester, Chester, United Kingdom; University of the Balearic Islands, SPAIN

## Abstract

**Background:**

Excess adiposity is associated with cardiovascular disease (CVD) risk factors such as hypertension, diabetes mellitus and dyslipidemia. Amongst the various measures of adiposity, the best one to help predict these risk factors remains contentious. A novel index of adiposity, the Body Adiposity Index (BAI) was proposed in 2011, and has not been extensively studied in all populations. Therefore, the purpose of this study is to compare the relationship between Body Mass Index (BMI), Waist Circumference (WC), Waist-to-Hip Ratio (WHR), Waist-to-Height Ratio (WHtR), Body Adiposity Index (BAI) and CVD risk factors in the local adult population.

**Methods and Findings:**

This is a cross sectional study involving 1,891 subjects (Chinese 59.1% Malay 22.2%, Indian 18.7%), aged 21–74 years, based on an employee health screening (2012) undertaken at a hospital in Singapore. Anthropometric indices and CVD risk factor variables were measured, and Spearman correlation, Receiver Operating Characteristic (ROC) curves and multiple logistic regressions were used. BAI consistently had the lower correlation, area under ROC and odd ratio values when compared with BMI, WC and WHtR, although differences were often small with overlapping 95% confidence intervals. After adjusting for BMI, BAI did not further increase the odds of CVD risk factors, unlike WC and WHtR (for all except hypertension and low high density lipoprotein cholesterol). When subjects with the various CVD risk factors were grouped according to established cut-offs, a BMI of ≥23.0 kg/m^2^ and/or WHtR ≥0.5 identified the highest proportion for all the CVD risk factors in both genders, even higher than a combination of BMI and WC.

**Conclusions:**

BAI may function as a measure of overall adiposity but it is unlikely to be better than BMI. A combination of BMI and WHtR could have the best clinical utility in identifying patients with CVD risk factors in an adult population in Singapore.

## Introduction

The prevalence of obesity in the world has risen to epidemic proportions [1]. This is of major concern as excess adiposity is strongly associated with cardiovascular disease (CVD) risk factors such as hypertension, diabetes mellitus and dyslipidemia [2,3]. Hence, a simple and effective measure of adiposity is needed for risk assessment in order to guide appropriate management and develop preventive strategies. However, this ‘best’ measure of adiposity to help predict these CVD risk factors remains contentious despite years of research.

Body Mass Index (BMI) is the widely used measure of obesity. However, the BMI is unable to differentiate between lean mass and fat mass, and hence, it is limited by differences in body adiposity for a given BMI across age, gender and ethnicity [4]. For example, the current definition of obesity based on BMI (BMI ≥30 kg/m^2^) may actually underestimate obesity among non-Caucasian populations, especially Asians [5]. In addition, the BMI does not consider body fat distribution, which is an important limitation since there are suggestions that the metabolic complications of obesity are more closely related to visceral adiposity than overall adiposity [2]. Hence, other measures of adiposity, which consider body fat distribution, like waist circumference (WC), waist-to-hip ratio (WHR) and waist-to-height ratio (WHtR) have been developed and studied. WC has been proposed to be the best amongst these measures, with excellent correlation with abdominal imaging and high association with CVD risk factors, especially diabetes [2,6,7]. However, WC does not account for differences in height, therefore, potentially over- and under-evaluating risk for tall and short individuals respectively [8]. Consequently, several researchers independently proposed the WHtR as an alternative to WC. This ratio has been shown to be a good indicator of abdominal adiposity, similar to WC [9] and recent systematic reviews and meta-analyses have supported the use of WHtR as a better predictor of CVD risk factors [8,10–12].

In 2011, the Body Adiposity Index (BAI) was proposed [13]. This is a composite index based on hip circumference and height (BAI = hip circumference (cm) divided by (height (m))^1.5^ minus 18), and was developed with the intention that this index would provide a direct estimate of percentage (%) body adiposity. From a population study of Mexican Americans, it was found that hip circumference (positive correlation) and height (negative correlation) were the most correlated variables with dual energy X-ray absorptiometry (DEXA)-derived % body adiposity. The final formula was derived to predict DEXA-derived % body adiposity, thus overcoming the limitation of the BMI in differentiating between fat and lean mass. This new index was then validated in a separate study of African Americans, and hence was suggested as a ‘better index of body adiposity by Bergman et al. [13]. However, validation (of the BAI) studies done in various populations with different ethnicities have shown consistently that the BAI tends to overestimate adiposity at lower percentage body fat (%BF) and underestimate adiposity at higher %BF [14]. Additionally, although the BAI seemed to correlate with DEXA-derived % adiposity better than the BMI when males and females were considered together, these studies showed that this was no longer the case when stratified by gender [14]. Nonetheless, excess adiposity as determined by %BF would theoretically predict cardiovascular disease and its risk factors better than indirect indices.

As mentioned, the best adiposity measure to help predict CVD risk factors has remained contentious. Although there are systematic reviews and meta-analyses that consistently support the case for WHtR, these reviews and meta-analyses tend to have more Asians than Caucasians [10,12], with subgroup analyses showing more positive results for WHtR in the Asian group than the Caucasian group [12]. This would be consistent with the findings by individual studies in Western populations, showing that WC is the better adiposity measure in predicting CVD risk factors [15–17] though some have continued to stress the importance of BMI [18,19]. Additionally, a systematic review and meta-analysis done exclusively on the Caucasian population, which included WHtR as a comparator, concluded that WC was more associated with CVD risk factors, and therefore recommended the use of WC in the clinic and in research studies [20].

Thus, further research is warranted in population groups and ethnicities where the various anthropometric measures including the recently proposed BAI have not been extensively analyzed and compared. Therefore, the purpose of this study is to compare the relationship between various anthropometric measures (BMI, WC, WHR, WHtR and the BAI) and CVD risk factors (i.e. hypertension, diabetes mellitus and dyslipidemia) in an adult population in Singapore, a South-East Asian country. The aims of the study are, firstly, to evaluate the performance of the BAI as a measure of adiposity, and secondly, to evaluate if WHtR is indeed a better predictor of CVD risk factors in this population.

## Methods

### Study Subjects

This was a retrospective study based on existing data, which were derived from standard procedures during an annual employee health screening conducted in 2012, and de-identified prior to data analysis. These subjects were employees from Khoo Teck Puat Hospital (KTPH), a regional hospital in Singapore. Only those who were 21 years old and above, and of Chinese, Malay or Indian ethnicity were considered for this study, yielding a sample of 1,945 subjects. Of these, subjects with incomplete and/or unverifiable data for the health screening questionnaire or study measurements were excluded (n = 54), resulting in a final sample of 1,891 subjects for analysis. No new data were collected (thus, there was no consent taking process in this study), and the data were collected and analyzed in a manner that subjects cannot be identified, directly or through identifiers linked to the subjects. Hence, this research involved no more than minimal risk to the individual, and the study proceeded only after obtaining approval from the National Healthcare Group Domain Specific Review Board (NHG DSRB, Reference Number: 2013/00598).

### Health Screening Questionnaire

During the health screening, conducted in KTPH, participants were required to complete a questionnaire to determine their demographic characteristics and past medical history of hypertension, diabetes and dyslipidemia, including treatments, if any, for these conditions. ‘Smokers’ were defined as those who were currently smoking either regularly or occasionally and ‘regular exercise’ was defined as 150 minutes of moderate-intensity aerobic activity a week or 60 minutes of vigorous-intensity aerobic activity a week.

### Health Screening Measurements

Well-trained examiners measured anthropometric indices, with participants wearing only minimal clothing with no footwear during measurements. Weight and height were measured, with the subject standing, to the nearest 0.1 kg and 1 cm, respectively, using a strain gauge scale, which was also equipped with a stadiometer (Seca 769, Hamburg, Germany). BMI was calculated in the standard way: weight (kg) divided by square of height (m). Waist and hip circumferences were measured to the nearest 0.1 cm using a flexible metric measuring tape with the subject in a standing position. Waist circumference was measured around the abdomen at the level of the umbilicus. Hip circumference was measured at the level of the maximum extension of the buttocks posteriorly in a horizontal plane. WHR was calculated as waist circumference (cm) divided by hip circumference (cm), while WHtR was calculated as waist circumference (cm) divided by height (cm). BAI was calculated as proposed by Bergman et al. [13]: hip circumference (cm) divided by (height (m))^1.5^ minus 18. Resting blood pressure (BP) was measured after five minutes in a seated position with an automated BP monitor using the oscillometric method (Omron HEM-7211, Kyoto, Japan). Any systolic BP found to be ≥140mmHg and/or diastolic BP ≥90mmHg was verified with a standard mercury sphygmomanometer after a two-minute interval.

### Laboratory Analysis

Blood samples were collected on-site, with the subjects having fasted for at least 8 hours, and sent to an internationally certified laboratory located within the hospital. Fasting blood glucose (FBG) was measured quantitatively by the enzymatic reference method with hexokinase [21] while total cholesterol (TC), high-density lipoprotein cholesterol (HDL-C) and triglycerides (TG) were assessed using standard enzymatic colorimetric method [22,23]. Low-density lipoprotein cholesterol (LDL-C) was estimated indirectly using the Friedwald formula [24]: LDL-C = TC-(HDL-C+(TG/5)) for subjects with TG levels <400 mg/dl. All samples were analyzed with an auto-analyzer (cobas 501, Roche, Basel, Switzerland).

### Definition of CVD Risk Factors

Hypertension was defined as having one or more of the following: (1) a systolic BP ≥140 mmHg, (2) a diastolic BP ≥90 mmHg, (3) physician-diagnosed hypertension and (4) use of antihypertensive medication. Diabetes mellitus was defined as having one or more of the following: (1) FBG ≥126 mg/dl, (2) physician-diagnosed diabetes mellitus and (3) use of oral hypoglycemic agents. The cut-off points for dyslipidemia were plasma TC ≥240 mg/dl and/or use of medications to lower blood cholesterol for high TC, TG ≥200 mg/dl for high TG, HDL-C <40 mg/dl for low HDL-C, and LDL-C ≥160 mg/dl and/or use of medications to lower blood cholesterol for high LDL-C [25].

### Statistical Analysis

All statistical analyses were performed using SPSS version 21 (SPSS Inc., Chicago, IL, USA). Continuous variables were tested for normality using the Kolmogorov-Smirnov test. Comparisons between males and females were performed using two independent samples t-test or the Mann-Whitney U test (as appropriate) for continuous and the chi-square test for categorical data. The relationship between anthropometric measures and CVD risk factors was first examined using Spearman’s correlation analysis. Receiver Operating Characteristic (ROC) analyses were then used to calculate the area under ROC curves (AUC) between each CVD risk factor and anthropometric measure, adjusted for age, gender, ethnicity, smoking status and physical activity status. The ROC was also used to identify cut-off values that best balanced sensitivity and specificity for the anthropometric measure with regards to the specific CVD risk factor, with respective sensitivity and specificity values reported. If multiple cut-off values were generated, the highest value is the one presented. Additionally, sensitivity and specificity based on established cut-off values for the various anthropometric measures were also explored using the ROC curves. Next, multiple logistic regression was used to evaluate the association between CVD risk factor and each standard deviation (SD) increase of the anthropometric measure adjusted for age, gender, ethnicity, smoking status and physical activity status. The effect of central obesity on overall obesity was also examined using multivariate models. Finally, in an attempt to delineate the clinical utility of BMI, WC and WHtR, grouping patients based on established cut off for BMI (≥23.0 kg/m^2^) [5], WC (≥80 cm for females, ≥90 cm for males) [26], and WHtR (≥0.5) [8], was done. All analyses were two-tailed, and a P value of <0.05 was considered to indicate statistical significance.

## Results

### Characteristics of the Study Subjects

The basic characteristics of the study population, stratified by gender, are shown in Table 1. 78.1% (n = 1,476) of the subjects were females, and the mean age of the study population was 35.7 years, with a mean BMI of 23.2 kg/m^2^ (minimum: 13.8 kg/m^2^, maximum: 40.9 kg/m^2^). The majority of the subjects was Chinese (n = 1,118, 59.1%) with the rest being Malay (n = 420, 22.2%) or Indian (n = 353, 18.7%). Males tended to have higher mean height, weight, waist circumference, hip circumference, BMI, WHR, and WHtR, but lower mean BAI (all P<0.001).

**Table 1:** Characteristics of study subjects.

Characteristics	Total (n = 1891)	Male (n = 415)	Female (n = 1476)	P-value*
**Ethnicity**				**0.003**
Chinese, n (%)	1118 (59.1)	273 (65.8)	845 (57.2)	
Malay, n (%)	420 (22.2)	69 (16.6)	351 (23.8)	
Indian, n (%)	353 (18.7)	73 (17.6)	280 (19.0)	
**Lifestyle factors**				
Smoker, n (%)	70 (3.7)	35 (8.4)	35 (2.4)	**<0.001**
Regular Exercise, n (%)	1013 (53.6)	280 (67.5)	733 (49.7)	**<0.001**
**Age (years)**	35.7 (±12.1)	39.0 (±12.1)	34.7 (±11.9)	**<0.001**
**Anthropometric measures**				
Height (m)	1.61 (±0.08)	1.70 (±0.07)	1.58 (±0.06)	**<0.001**
Weight (kg)	60.1 (±12.6)	69.8 (±13.1)	57.3 (±11.0)	**<0.001**
Waist (cm)	77.9 (±11.2)	85.1 (±10.5)	75.8 (±10.6)	**<0.001**
Hip (cm)	95.1 (±8.9)	96.8 (±8.4)	94.6 (±9.0)	**<0.001**
**Anthropometric indices**				
Body Mass Index (BMI) (kg/m^2^)	23.2 (±4.2)	24.1 (±4.1)	22.9 (±4.2)	**<0.001**
Waist Hip Ratio (WHR)	0.82 (±0.08)	0.88 (±0.06)	0.80 (±0.07)	**<0.001**
Waist Height Ratio (WHtR)	0.48 (±0.07)	0.50 (±0.06)	0.48 (±0.07)	**<0.001**
Body adiposity index (BAI) (%)	28.7 (±5.0)	25.7 (±4.1)	29.6 (±4.9)	**<0.001**
**CVD RF measurements**				
Fasting Glucose (mg/dl)^†^	88.3 (82.9–95.5)	91.9 (86.5–99.1)	88.3 (82.9–93.7)	**<0.001**
Systolic Blood Pressure (mm Hg)	117 (±14.4)	126 (±13.0)	114 (±13.7)	**<0.001**
Diastolic Blood Pressure (mm Hg)	75 (±9.5)	79 (±9.4)	74 (±9.1)	**<0.001**
Total Cholesterol (mg/dl)	201.9 (±36.0)	205.8 (±38.1)	200.8 (±35.3)	**0.012**
Triglycerides^†^ (mg/dl)	73.5 (54.9–104.5)	92.1 (65.5–132.9)	70.0 (53.1–96.5)	**<0.001**
HDL Cholesterol (mg/dl)	65.3 (±16.2)	56.7 (±14.0)	67.7 (±15.9)	**<0.001**
LDL Cholesterol (mg/dl)	119.3 (±32.7)	127 (±34.4)	117.0 (±31.8)	**<0.001**
**Prevalence of CVD RF, n (%)**				
Diabetes Mellitus	94 (5.0)	29 (7.0)	65 (4.4)	**0.032**
Hypertension	257 (13.6)	111 (26.7)	146 (9.9)	**<0.001**
High Total Cholesterol	402 (21.3)	107 (25.8)	295 (20.0)	**0.011**
High Triglycerides	72 (3.8)	34 (8.2)	38 (2.6)	**<0.001**
Low HDL-C	72 (3.8)	43 (10.4)	29 (2.0)	**<0.001**
High LDL-C	355 (18.8)	105 (25.3)	250 (16.9)	**<0.001**

*P-value from Mann-Whitney U test for Fasting Glucose and Triglycerides, two independent samples t-test for all other continuous variables, and chi-square test for categorical variables. These tests were done to compare between males and females.

^†^Median (Interquartile range) presented

Mean (± standard deviation) presented unless otherwise stated

CVD RF: Cardiovascular disease risk factors

HDL, high-density lipoprotein; LDL, low-density lipoprotein.

As for CVD risk factor variables, males tended to have higher median FBG (P<0.001) and TG level (P<0.001), higher mean systolic and diastolic BP (both P<0.001), TC level (P = 0.012), and LDL-C level (P<0.001) but lower mean HDL-C level (P<0.001). Correspondingly, the percentages of males with diabetes mellitus, hypertension, high TC, high TG, low HDL-C and high LDL-C were all significantly higher than the percentages in females (all P<0.05).

### Correlations between Anthropometric Measures and CVD Risk Factor Variables

The Spearman’s correlation coefficients between the various anthropometric measures with the CVD risk factor variables, stratified by gender, are shown in Table 2. All the anthropometric measures correlated significantly with CVD risk factor variables, with BAI consistently having the lowest correlation coefficients when both genders were considered together. In this same scenario, measures of central adiposity, namely WC and WHtR, correlated best with the various CVD risk factor variables, with WC correlating the best for five out of the seven CVD risk factor variables (FBG, systolic and diastolic BP, TG level and HDL-C level). When genders were considered separately, measures of central adiposity (WC and WHtR) continued to correlate the best with CVD risk factor variables in males, while BMI (measure of overall adiposity) became the best correlated for three out of the seven CVD risk factor variables (systolic and diastolic BP, and HDL-C level) in females with WHtR correlating the best for the rest of the variables.

**Table 2:** Spearman Correlation Coefficient between Anthropometric Indices and Cardiovascular Disease Risk Factors.

	Body Mass Index	Waist Circumference	Waist-Hip Ratio	Waist-Height Ratio	Body Adiposity Index
**Overall (n = 1891)**					
Fasting Glucose	0.335	**0.368**	0.326	0.366	0.197
Systolic Blood Pressure	0.446	**0.472**	0.364	0.408	0.195
Diastolic Blood Pressure	0.380	**0.397**	0.310	0.350	0.174
Total Cholesterol	0.174	0.198	0.178	**0.213**	0.142
Triglycerides	0.399	**0.456**	0.406	0.443	0.224
HDL Cholesterol	-0.437	**-0.470**	-0.370	-0.433	0.220
LDL Cholesterol	0.300	0.322	0.265	**0.327**	0.209
**Male (n = 415)**					
Fasting Glucose	0.234	0.295	0.324	**0.333**	0.242
Systolic Blood Pressure	0.357	**0.359**	0.220	0.325	0.274
Diastolic Blood Pressure	0.343	**0.363**	0.253	0.340	0.272
Total Cholesterol	0.166	0.190	0.182	**0.205**	0.188
Triglycerides	0.414	**0.440**	0.365	0.430	0.333
HDL Cholesterol	-0.411	**-0.424**	-0.318	-0.423	-0.334
LDL Cholesterol	0.211	0.229	0.208	**0.249**	0.227
**Female (n = 1476)**					
Fasting Glucose	0.333	0.340	0.278	**0.349**	0.279
Systolic Blood Pressure	**0.438**	0.396	0.234	0.387	0.376
Diastolic Blood Pressure	**0.359**	0.333	0.218	0.320	0.287
Total Cholesterol	0.168	0.187	0.173	**0.205**	0.172
Triglycerides	0.369	0.405	0.342	**0.418**	0.322
HDL Cholesterol	**-0.416**	-0.409	-0.271	-0.410	-0.358
LDL Cholesterol	0.305	0.307	0.234	**0.327**	0.295

All are significant at the level of <0.01 (2-tailed).

Anthropometric measure with highest correlation coefficient for each variable in **bold**.

HDL, high-density lipoprotein; LDL, low-density lipoprotein.

### Association of Various Anthropometric Measures and CVD Risk Factors using ROC Curve Analyses

The area under ROC curves (AUC) between each CVD risk factor and anthropometric measure, after adjusting for age, gender, ethnicity, smoking status and physical activity status, are shown in Table 3. WC had the largest AUC for four out of the six CVD risk factors (diabetes mellitus, high TC, high TG, and high LDL-C), while BMI had the largest AUC for the other two (hypertension and low HDL-C). However, the differences in the AUC for the various anthropometric measures were often small with overlapping 95% confidence intervals (CIs).

**Table 3:** Adjusted Area Under Receiver Operating Characteristic (ROC) Curve for the Various Anthropometric Indices and Cardiovascular Disease Risk Factors.

	Body Mass Index	Waist Circumference	Waist-Hip Ratio	Waist-Height Ratio	Body Adiposity Index
**Diabetes mellitus**	0.871 (0.841–0.900)	**0.874 (0.842–0.907)**	0.862 (0.824–0.900)	0.871 (0.838–0.904)	0.849 (0.814–0.884)
**Hypertension**	**0.862 (0.840–0.883)**	0.854 (0.831–0.876)	0.834 (0.809–0.858)	0.854 (0.832–0.877)	0.846 (0.823–0.869)
**High Total Cholesterol**	0.766 (0.739–0.792)	**0.771 (0.745–0.797)**	0.768 (0.741–0.794)	0.770 (0.744–0.796)	0.759 (0.732–0.786)
**High Triglyceride**	0.823 (0.779–0.866)	**0.826 (0.782–0.869)**	0.775 (0.726–0.825)	0.822 (0.780–0.864)	0.788 (0.742–0.834)
**Low HDL-Cholesterol**	**0.853 (0.816–0.891)**	0.844 (0.805–0.883)	0.810 (0.765–0.854)	0.844 (0.805–0.882)	0.827 (0.787–0.868)
**High LDL-Cholesterol**	0.766 (0.739–0.794)	**0.770 (0.742–0.797)**	0.766 (0.738–0.794)	**0.770 (0.743–0.798)**	0.759 (0.731–0.787)

Anthropometric measure with the highest AUC value in **bold**.

HDL, high-density lipoprotein; LDL, low-density lipoprotein.

The optimal cut-off points that best balanced sensitivity and specificity for the various CVD risk factors of BMI, WC and WHtR, in males and females, are shown in Table 4a. The cut off values in males ranged from 23.5 kg/m^2^ to 23.9 kg/m^2^ for BMI, 84.5 cm to 88.5 cm for WC and 0.479 to 0.501 for WHtR; and in females, from 23.5 kg/m^2^ to 27.7 kg/m^2^ for BMI, 79.5 cm to 83.5 cm for WC and 0.460 to 0.531 for WHtR. When cut off points were based on recommended cut-offs for the Asian population for BMI (high risk: BMI ≥27.5 kg/m^2^) and WC (≥80 cm for females, ≥90 cm for males), and the proposed cut off of 0.5 for WHtR, WC and WHtR (measures of central adiposity) had better sensitivities compared to BMI, while BMI (of 27.5 kg/m^2^) had the highest specificities, in both genders (Table 4b). Comparing between WC (≥80 cm for females, ≥90 cm for males) and WHtR (0.5) only, WHtR in general had slightly better sensitivities (except for low HDL-C in males and females, and low LDL-C in males), while WC had generally better specificities (except for low HDL-C in males and females, and low LDL-C in males).

**Table 4:** Cut-Off Points, Sensitivity and Specificity for BMI, WC and WHtR Predictive of Cardiovascular Disease Risk Factors.

a. Optimal Cut-Off Points for BMI, WC and WHtR Predictive of CVD Risk Factors									
	Body Mass Index (BMI)			Waist Circumference (WC)			Waist-Height Ratio (WHtR)		
	Cut-off (kg/m^2^)	Sensitivity (%)	Specificity (%)	Cut-off (cm)	Sensitivity (%)	Specificity (%)	Cut-off	Sensitivity (%)	Specificity (%)
**Male (n = 415)**									
Diabetes mellitus	23.9	69.0	56.5	88.5	69.0	69.2	0.491	96.6	49.7
Hypertension	23.8	66.7	60.5	84.5	72.1	56.9	0.501	67.6	61.8
High Total Cholesterol	23.5	67.3	58.1	86.5	60.7	65.6	0.479	83.2	46.1
High Triglyceride	23.7	82.4	55.9	88.5	70.6	69.8	0.492	82.4	51.4
Low HDL-Cholesterol	23.5	83.7	53.8	88.5	67.4	70.4	0.491	90.7	50.8
High LDL-Cholesterol	23.5	67.6	58.1	86.5	60.0	65.2	0.479	83.8	46.1
**Female (n = 1476)**									
Diabetes mellitus	27.1	49.2	85.5	82.5	72.3	76.8	0.513	80.0	72.3
Hypertension	27.7	41.1	42.5	83.5	61.6	81.5	0.531	61.0	81.6
High Total Cholesterol	23.6	53.2	67.0	79.5	53.2	70.4	0.460	75.9	48.5
High Triglyceride	25.6	76.3	78.4	83.5	76.3	78.7	0.523	81.6	75.7
Low HDL-Cholesterol	26.1	75.9	80.1	78.8	79.3	63.3	0.497	79.3	62.8
High LDL-Cholesterol	23.5	57.6	66.5	79.5	60.0	70.9	0.499	61.6	68.7
b. Sensitivity and Specificity for BMI, WC and WHtR Predictive of CVD Risk Factors Based On Established/Proposed Cut-Off Points*									
	**Body Mass Index (BMI)**			**Waist Circumference (WC)**			**Waist-Height Ratio (WHtR)**		
	**Cut-off (kg/m** ^**2**^ **)**	**Sensitivity (%)**	**Specificity (%)**	**Cut-off (cm)**	**Sensitivity (%)**	**Specificity (%)**	**Cut-off**	**Sensitivity (%)**	**Specificity (%)**
**Male (n = 415)**									
Diabetes mellitus	27.5	24.1	82.1	90	62.1	72.8	0.501	79.3	56.5
Hypertension	27.5	31.5	86.5	90	44.1	75.7	0.501	67.6	61.8
High Total Cholesterol	27.5	26.2	84.4	90	42.1	74.7	0.501	63.6	60.1
High Triglyceride	27.5	41.2	83.7	90	61.8	73.2	0.501	76.5	56.7
Low HDL-Cholesterol	27.5	44.2	84.7	90	95.3	33.6	0.501	76.7	57.5
High LDL-Cholesterol	27.5	25.7	84.2	90	85.7	36.1	0.501	64.8	60.3
**Female (n = 1476)**									
Diabetes mellitus	27.5	43.1	87.6	80	80.0	67.8	0.499	84.6	65.8
Hypertension	27.5	44.5	89.6	80	69.2	69.5	0.499	72.6	67.5
High Total Cholesterol	27.5	22.7	88.5	80	53.2	70.4	0.499	55.6	68.3
High Triglyceride	27.5	42.1	87.0	80	84.2	67.0	0.499	84.2	64.8
Low HDL-Cholesterol	27.5	34.5	86.7	80	75.9	33.5	0.499	72.4	64.3
High LDL-Cholesterol	27.5	25.2	88.6	80	60.0	70.9	0.499	61.6	68.7

*BMI ≥27.5 kg/m^2^ (High Risk Category, Obesity equivalent for Asians)

WC ≥80cm for females, WC ≥90cm for males (Asian Cut-Offs); WHtR ≥0.5 (Proposed Cut Off)

HDL, high-density lipoprotein; LDL, low-density lipoprotein.

### Odds Ratios of CVD Risk Factors by Anthropometric Measures

Multivariate-adjusted odds ratios (ORs) of each CVD risk factor with each SD increase of the anthropometric measure are shown in Tables 5 and 6. In the first model, the ORs were adjusted for age, gender, ethnicity, smoking status and physical activity status, and this showed that each SD increase of WC had the highest ORs for all the CVD risk factors amongst all the anthropometric measures (Table 5a), with the OR of high TG being significantly higher for each SD increase of WC (2.78, 95% CI: 2.13–3.61) than for each SD increase of WHR (1.60, 95% CI: 1.25–2.05). However, the rest of the ORs were not considered significantly different from each other due to the overlapping 95% confidence intervals. In the second model, adjustment for BMI was added into the model, and this showed that each SD increase of WC continued to have significant ORs for diabetes mellitus, high TC, high TG and high LDL-C, with WC having the highest OR for all except high LDL-C (WHtR had the highest OR) (Table 5b). Comparing the ORs between WC and WHtR in this model, they are not significantly different except for high TG where each SD increase of WC still had a significant OR (2.00, 95% CI: 1.21–3.30) whereas each SD increase of WHtR became non-significant (1.61, 95% CI: 0.99–2.63).

**Table 5:** Adjusted Odds Ratios of Cardiovascular Disease Risk Factors by Anthropometric Indices.

a. Model 1. Odds Ratio Adjusted for Age, Gender, Ethnicity, Smoking Status and Physical Activity Status					
	Body Mass Index	Waist Circumference	Waist-Hip Ratio	Waist-Height Ratio	Body Adiposity Index
Diabetes mellitus	1.91 (1.54–2.36)***	2.26 (1.75–2.93)***	1.95 (1.50–2.52)***	2.13 (1.66–2.73)***	1.43 (1.13–1.81)**
Hypertension	2.27 (1.94–2.66)***	2.31 (1.94–2.76)***	1.53 (1.28–1.82)***	2.30 (1.93–2.75)***	2.08 (1.74–2.48)***
High Total Cholesterol	1.37 (1.21–1.55)***	1.52 (1.32–1.75)***	1.45 (1.25–1.68)***	1.49 (1.30–1.72)***	1.25 (1.09–1.43)**
High Triglyceride	2.28 (1.84–2.82)***	2.78 (2.13–3.61)***	1.60 (1.25–2.05)***	2.58 (2.00–3.33)***	2.11 (1.64–2.72)***
Low HDL-Cholesterol	2.14 (1.71–2.68)***	2.26 (1.74–2.94)***	1.46 (1.15–1.84)**	2.19 (1.69–2.82)***	1.89 (1.46–2.46)***
High LDL-Cholesterol	1.42 (1.25–1.61)***	1.58 (1.37–1.83)***	1.53 (1.31–1.79)***	**1.58 (1.37–1.83)** ***	1.31 (1.14–1.51)***
**b**. **Model 2**. Odds Ratio Adjusted for Age, Gender, Ethnicity, Smoking Status, Physical Activity Status and Body Mass Index					
	**Body Mass Index**	**Waist Circumference**	**Waist-Hip Ratio**	**Waist-Height Ratio**	**Body Adiposity Index**
Diabetes mellitus	-	1.80 (1.15–2.83)*	1.67 (1.27–2.20)***	1.59 (1.02–2.48)*	0.65 (0.46–0.91)*
Hypertension	-	1.26 (0.92–1.71)	1.13 (0.94–1.35)	1.26 (0.92–1.72)	0.99 (0.75–1.30)
High Total Cholesterol	-	1.48 (1.16–1.90)**	1.32 (1.13–1.55)**	1.43 (1.12–1.83)**	0.86 (0.69–1.08)
High Triglyceride	-	2.00 (1.21–3.30)**	1.24 (0.97–1.58)	1.61 (0.99–2.63)	0.91 (0.61–1.35)
Low HDL-Cholesterol	-	1.28 (0.77–2.11)	1.15 (0.90–1.47)	1.15 (0.71–1.88)	0.84 (0.56–1.24)
High LDL-Cholesterol	-	1.52 (1.18–1.96)**	1.38 (1.17–1.63)***	1.56 (1.21–2.02)**	0.91 (0.72–1.14)

Anthropometric measure with the highest significant OR value in **bold**

*P<0.05

**P<0.01

***P<0.001

HDL, high-density lipoprotein; LDL, low-density lipoprotein.

**Table 6:** Odds Ratio of Cardiovascular Disease Risk Factors Adjusted for Age, Gender, Ethnicity, Smoking Status, Physical Activity Status and Waist Circumference or Waist-Height Ratio (Model 3).

	Body Mass Index^†^	Body Mass Index^‡^
Diabetes mellitus	1.27 (0.86–1.86)	1.36 (0.92–2.01)
Hypertension	1.92 (1.46–2.53)***	1.91 (1.44–2.53)***
High Total Cholesterol	1.03 (0.83–1.28)	1.05 (0.84–1.31)
High Triglyceride	1.39 (0.91–2.12)	1.60 (1.05–2.45)*
Low HDL-Cholesterol	1.79 (1.15–2.77)**	1.93 (1.25–2.97)**
High LDL-Cholesterol	1.05 (0.83–1.31)	1.02 (0.91–1.64)

*P<0.05

**P<0.01

***P<0.001

^†^ Adjusted for Age, Gender, Ethnicity, Smoking Status, Physical Activity Status and Waist Circumference

^‡^ Adjusted for Age, Gender, Ethnicity, Smoking Status, Physical Activity Status and Waist-Height Ratio

HDL, high-density lipoprotein; LDL, low-density lipoprotein.

Finally, when adjustment for WC or WHtR was added into the model, each SD increase of BMI still had significant ORs for hypertension and low HDL-C (Table 6).

### Proportion of Subjects Identified Based on Established/Proposed Cut Offs for BMI, WC and WHtR for Each CVD Risk Factor Group

The proportion of those who would have been identified based on established cut offs for BMI (≥23.0 kg/m^2^), WC (≥80 cm for females, ≥90 cm for males), and WHtR (≥0.5), singly or in combination, are shown in Table 7. Based on a WHtR of ≥0.5, the proportion of those who would have been identified was consistently higher than that for a WC of ≥80 cm in females, and WC ≥90 cm in males, for all CVD risk factors. When a combination of measures were included in the evaluation, a BMI of ≥23.0 kg/m^2^ and/or WHtR ≥0.5 identified the highest proportion for all the CVD risk factors in both genders, even higher than a combination of BMI and WC.

**Table 7:** Proportion of Subjects with Cardiovascular Disease Risk Factors Stratified by Gender Based on Established/Proposed Cut Offs for Body Mass Index*, Waist Circumference^†^ and Waist-Height Ratio^‡^.

	Males, n(%)						Females, n(%)					
	Hypertension (n = 111)	Diabetes Mellitus (n = 29)	High Total Cholesterol (n = 107)	High Triglycerides (n = 34)	Low HDL Cholesterol (n = 43)	High LDL Cholesterol (n = 105)	Hypertension (n = 146)	Diabetes Mellitus (n = 65)	High Total Cholesterol (n = 295)	High Triglycerides (n = 38)	Low HDL Cholesterol (n = 29)	High LDL Cholesterol (n = 250)
**Alone**												
**Body Mass Index (BMI), kg/m** ^**2**^												
BMI<23.0	30(27.0)	7(24.1)	30(28.0)	5(14.7)	6(14.0)	27(25.7)	32(21.9)	10(15.4)	126(42.7)	6(15.8)	6(20.7)	96(38.4)
BMI≥23.0	81(73.0)	22(75.9)	77(72.0)	29(85.3)	37(86.0)	78(74.3)	114(78.1)	55(84.6)	169(57.3)	32(84.2)	23(79.3)	154(61.6)
**Waist Circumference (WC), cm**												
WC<80,90^†^	62(55.9)	11(37.9)	62(57.9)	13(38.2)	16(37.2)	61(58.1)	45(30.8)	13(20.0)	138(46.8)	6(15.8)	7(24.1)	100(40.0)
WC≥80,90^†^	49(44.1)	18(62.1)	45(42.1)	21(61.8)	27(62.8)	44(41.9)	101(69.2)	52(80.0)	157(53.2)	32(84.2)	22(75.9)	150(60.0)
**Waist-to-Height Ratio (WHtR)**												
WHtR<0.5	33(29.7)	3(10.3)	34(31.8)	7(20.6)	7(16.3)	32(30.5)	39(26.7)	9(13.8)	128(43.4)	5(13.2)	6(20.7)	94(37.6)
WHtR>0.5	78(70.3)	26(89.7)	73(68.2)	27(79.4)	36(83.7)	73(69.5)	107(73.3)	56(86.2)	167(56.6)	33(86.8)	23(79.3)	156(62.4)
**In Combination**												
**BMI, kg/m** ^**2**^ **and WC, cm**												
BMI<23.0 and WC<80,90^†^	28(25.2)	6(20.7)	29(27.1)	5(14.7)	6(14.0)	26(24.8)	28(19.2)	7(10.8)	104(35.3)	4(10.5)	4(13.8)	76(30.4)
BMI≥23.0 and/or WC≥80,90^†^	83(74.8)	23(79.3)	78(72.9)	29(85.3)	37(86.0)	79(75.2)	118(80.8)	58(89.2)	191(64.7)	**34(89.5)**	25(86.2)	174(69.6)
**BMI, kg/m** ^**2**^ **and WHtR**												
BMI<23.0 and WHtR<0.5	23(20.7)	2(6.7)	22(20.6)	4(11.8)	4(9.3)	19(18.1)	26(17.8)	5(7.7)	100(33.9)	4(10.5)	3(10.3)	73(29.2)
BMI≥23.0 and/or WHtR≥0.5	**88(79.3)**	**27(93.1)**	**85(79.4)**	**30(88.2)**	**39(90.7)**	**86(81.9)**	**120(82.2)**	**60(92.3)**	**195(66.1)**	**34(89.5)**	**26(89.7)**	**177(70.8)**

Highest proportion in **bold**.

*BMI ≥ 23.0 kg/m^2^ (Increased Risk Category for Asians)

^†^WC ≥ 80cm for females, WC ≥ 90cm for males (Asian Cut-Offs)

^‡^WHtR ≥ 0.5 (Proposed Cut-Off)

HDL, high-density lipoprotein; LDL, low-density lipoprotein.

## Discussion

The first aim was to evaluate the utility of the recently proposed BAI as a measure of adiposity and the analyses reveal a few observations with regards to this. Firstly, when both genders were considered together, BAI consistently correlated the poorest with the various CVD risk factor variables amongst all the anthropometric measures being studied. Although BAI seemed to correlate better when stratified by gender, the correlation coefficients were generally lower than for WC and WHtR in males, and lower than for BMI and WHtR in females.

Secondly, based on adjusted AUCs between each CVD risk factor and the anthropometric measure, the BAI had consistently lower AUC values than BMI, WC and WHtR, although the differences were often small, with overlapping 95% confidence intervals. Thirdly, after adjusting for BMI, BAI did not further increase the odds of CVD risk factors unlike WC and WHtR, suggesting that BAI behaved similarly to BMI and hence, BAI has no additional value after taking BMI into account. This is consistent with the fact that although BAI attempts to give an estimation of %BF, it does not distinguish the distribution of that body adiposity, and thus would function as an overall measure of adiposity, like the BMI.

Hence, this study purports that the utility of the BAI would be that of an overall measure of adiposity like the BMI, and there is no suggestion that BAI would be better than the well-established BMI. Moreover, with validation studies consistently showing that the BAI tends to over- or underestimate adiposity at extreme ends of %BF, and that the BAI does not correlate with dual energy X-ray absorptiometer (DEXA)-derived % adiposity better than BMI when stratified by gender [14], the BAI is unlikely to be a better overall measure of adiposity than the BMI.

The second aim of this study was to evaluate if the WHtR is indeed a better predictor of CVD risk factors such as hypertension, diabetes mellitus and dyslipidemia in an adult population in Singapore, as suggested by recent systematic reviews and meta-analyses [8,10–12]. From the initial analyses of this study, it would seem that WC and WHtR (measures of central adiposity) are better than BMI (measure of overall adiposity) based on correlation coefficients, estimated AUC and OR values, with WC generally having the higher estimated values compared to WHtR. This is consistent with a number of studies concluding that measures of central adiposity such as WC is more closely associated with CVD risk factors than BMI [15,16,27–29], suggesting that the complications of obesity are more closely related to the distribution rather than the absolute degree of adiposity per se. However, it must be noted that the 95% confidence intervals of these AUC and OR values were all overlapping in this study, and hence, BMI, WC and WHtR could all be considered comparable in their association with CVD risk factors, which is consistent with some of the other studies as well [30–33].

When further analyses such as additional multiple regression models to investigate the effect of central adiposity on overall adiposity were done, it was found that each SD increase of WC continued to have significant ORs for diabetes mellitus, high TC, high TG and high LDL-C, after adjusting for BMI. This was similarly observed for WHtR (except for high TG where the OR became non-significant), suggesting that measures of central adiposity continue to increase the odds of these CVD risk factors, independent of BMI. As for hypertension and low HDL, the ORs for each SD increase of WC/WHtR became non-significant after accounting for BMI, suggesting that the association of WC and WHtR with hypertension and low HDL may not be independent of BMI. This is confirmed when WC or WHtR was included in the model, and each SD increase of BMI continued to have significant ORs, that is, the increase in odds is now independent of WC or WHtR. Other studies have made similar observations [34,35] and this is understood to be due to the BMI better reflecting body volume and mass, which is associated with blood viscosity and blood volume, and hence more closely related to BP, while measures of central adiposity are good indicators of visceral adiposity and hence more closely associated with diabetes [34].

Hence, this study suggests that using a combination of measures, one that includes a measure of general adiposity and a measure of central adiposity, would be more appropriate in the identifying of CVD risk factors, and this is supported by the observation that a BMI of ≥23.0 kg/m^2^ and/or WHtR ≥0.5 yielded the highest proportion who would be identified amongst those with the various CVD risk factors. For example, relying on a WC of ≥80 cm for females and ≥90cm for males would have identified 74.5% (70/94) of the people (males and females) with diabetes, while a combination of BMI ≥23.0 kg/m^2^ and/or WHtR ≥0.5 would have identified 92.6% (87/94), even more than a combination of BMI ≥23.0 kg/m^2^ and/or WC ≥80 cm for females, and ≥90 cm for males (86.2%, 81/94). This is consistent with this study’s finding that WHtR ≥0.5 was found to be generally more sensitive (based on ROC analysis) than WC ≥80 cm in females, and WC ≥90 cm in males, an attribute that is more desirable for a screening test since a diagnostic test (measurement of blood pressure or blood test) would still be required to confirm the diagnosis and rule the disease in. Thus, WHtR may have better utility than WC as a measure of obesity to screen for CVD risk factors.

In addition, unlike other indices of abdominal adiposity, a universal cut-off value for WHtR has been suggested in several studies, obviating the need for age-, sex- and ethnic-specific cut-off values [8,36]. In this study of an adult population in Singapore, the optimal cut off value found was 0.479 to 0.501 for males and 0.460 to 0.531 for females, approximating to the 0.5 proposed in several studies [8,36]. As an individual’s height is relatively fixed, the WHtR would allow individuals to have individualized cut offs for waist circumference while affording a simple public health message of getting the public to visit their doctors for a medical evaluation for CVD risk factors if their waist circumference is more than half their height, especially if their BMI is ≥23.0 kg/m^2^.

This study has important limitations. First, this study population (employees from a hospital) may not be entirely representative of the general adult Asian population in Singapore. Second, while the sample size is believed to be adequate for analysis as a whole, the small numbers of Malay and Indian prevented analyses stratified by ethnic groups. Hence, an approach to adjust for ethnic groups was used instead. We believe this approach allows for an overall conclusion in a multi-ethnic population, yet takes into account the effect of ethnicity on the relationship between measures of obesity and CVD risk factors. Third, age, gender, ethnicity, smoking and physical activity status have been associated with the development of CVD risk factors [26,37,38]. While these have been accounted for by the adjustment for these factors in the analysis, other factors known to influence the development of CVD risk factors such as dietary habits and family history have not been accounted for. Fourth, the findings of this cross-sectional study are not conclusive evidence of a causal relation of the various anthropometric measures with CVD risk factors. Further studies of a prospective nature would be required to confirm the findings of this study.

In conclusion, BAI may function as a measure of overall adiposity but it is unlikely to be better than BMI. While BMI, WC and WHtR seem comparable in their association with CVD risk factors, a combination of BMI and WHtR could have the best clinical utility in identifying patients with CVD risk factors in an adult population in Singapore.
